# Development of Carbon Nanostructured Based Electrochemical Sensors for Pharmaceutical Analysis

**DOI:** 10.22037/ijpr.2019.1100645

**Published:** 2019

**Authors:** Masoumeh Ghalkhani, Fatemeh Ghorbani-Bidkorbeh

**Affiliations:** a *Department of Chemistry, Faculty of Science, Shahid Rajaee Teacher Training University, Lavizan, Tehran, Iran. *; b *Department of Pharmaceutics, School of Pharmacy, Shahid Beheshti University of Medical Sciences, Tehran, Iran.*

**Keywords:** Pharmaceutical, Carbon nanostructures, Graphene, Carbon Nanotube, Nanoparticle, Voltammetric, Modified electrode

## Abstract

Pharmaceutical drugs play an important role in human life since they caused a revolution in human health. Notably, their administration to a living organism helps body to stay healthy. Commonly, they are employed to diagnose, prevent, or treat and cure a disease via a biological effect on a human body. Administration of impurity*-*free and adequate amounts of pharmaceutical compounds would be beneficial. Therefore, the quantity and purity of the substances in pharmaceutical compounds are continuously monitored during drug manufacturing with various chemical or instrumental analysis techniques. The possibility of impurities development and chemical or quantity changes of active drug species at various stages (namely during production, transportation or storage) makes them redundant and risky for human health. So, sensitive and accurate analysis methods for qualitative and quantitative analysis are highly demanded by pharmaceutical companies and medical centers. The present mini review emphasizes on application of carbon based modified electrodes in health care and pharmaceutical analysis. Electrochemical determination of drugs employing carbon nanostructured modified electrodes will be reviewed and their advantages and disadvantages will be mentioned.

## Introduction

Pharmaceutical analysis should be performed during all steps of pharmaceutical development (1-3), from the early stage of synthesis, formulation, stability testing and quality control to toxicological and pharmacological investigations in animals and humans in process development including preclinical and clinical trials (4, 5). After administration to patients, analytical measurements are essential for bioavailability testing and also for evaluating their effectiveness and investigation of the needed dosage of drug formulation (6, 7). Quantitative analysis of active ingredient content in drug formulations and also after application in human biological fluids is inevitable. While various analytical techniques have been developed for this aim, electrochemical techniques have been considered as sensitive, easy operation, efficient and cost effective methods which can be rapidly and accurately employed for pharmaceutical assay (7). Compared to other analytical methods, electrochemical determination has demonstrated to be highly sensitive and reliable with less interference from non-electro-active species for the determination of wide range of pharmaceutical agents (8, 9). Over the past two decades, electrochemical behaviour of drugs such as ascorbic acid, phenothiazines, benzodiazepines, paracetamol, tramadol, theophylline, sumatriptan, naltrexone, mercaptopurine, gabapentin, lamotrigine, etc. has been widely investigated and a great trend in pharmaceutical analysis can be seen for developing electrochemical sensors. In addition, intelligent drug delivery systems or implantable sensors for the rapid detection of biomarker and the release of therapeutic agents on demand can be designed based on these sensors (10).

*Development of modified electrodes*

Electrochemical techniques have been widely employed for both qualitative and quantitative analysis of organic (11-14) and inorganic (15, 16) compounds. In electrochemistry, working electrode surface plays an important role as the selectivity and sensitivity of the measurements is highly depended on its properties (17).

In voltammetry measurements, potential and current of resulted peak (s) in recorded voltammograms show two important characteristic of the target species. Peak potential specify the electro-active spices (18), while peak current is a function of the analyte concentration in the sample solution, effective surface area of the electrode and number of electrons transferred between analyte and electrode during redox reaction process (19). Notably, electrochemical response of most of the species is poor or insensible at low concentrations at ordinary bare electrodes such as carbon paste (20), glassy carbon (21), Pt (22, 23) or Au (24) electrodes. Chemically modified electrodes (CMEs) have attracted much interest in the construction of electro-chemical sensors as they can overcome mentioned intrinsic problems, and facilitate the interfacial electron transfer (25-28). Chemically modified electrodes are constructed by bonding or coating a thin film of mono or multi molecular, ionic or polymeric compounds onto the surface of a conducting or semiconducting substrate. The response of the modified electrodes exhibits the chemical or electrochemical properties of the modifier materials in the faradaic reactions. Here, enhancement of the desirable electrochemical properties and attenuation or elimination of the unwanted properties takes place. For a constant amount of analyte the peak current can be enhanced by increasing the electrode surface area such as surface modification with nanostructured materials (29). Also, surface modification can change the peak potential positively or negatively, depending on the modification process which can increase or decrease the over-potential of the redox reactions. This over-potential change can be related to changing the charge transfer way or electro-catalysis process or variation in mass transfer phenomena (30). A vast range of materials has been employed as electrode modifier toward improvement of sensitivity and selectivity of the electrochemical responses (20-22, 25-28 and 31-33). In recent decades, carbon nanostructured materials have well-played roles in electrochemistry. They are used as electrode modifier in a wide range of electrochemical experiments, from sensors (34-36) and biosensor (34, 37-39) to batteries (40), fuel cells (41), solar cells (42, 43), super capacitors (44, 45), and so on. Carbon nanostructures have attracted significant attention of researchers in the field of development of modified electrodes due to their unique properties such as high electrical conductivity, chemical stability, and very large surface area (46, 47). Many papers have been published on carbon nanostructured based modified electrodes (22, 47-49).

*Carbon nanostructured based electrode modification*

Various ways have been applied for electrode modification by carbon nanostructures. Generally, electrode modification includes bulk or surface modification. In simplest method, suspension of various types of carbon nanostructures is prepared in a suitable organic or inorganic solvent such as water, dimethyl form amide (DMF), or mixture of them (50, 51). Then, the optimized adequacy of this suspension was drop casted on the electrode surface, on which a thin layer of modifier is formed after solvent evaporation (50-53). Even though the operation of this method is very easy, the dispersion of some materials in applicable solvents is not possible. For example, strong interaction between multiwalled carbon nanotubes (MWCNTs) hinders their dispersion in any solvent. Notably, even after applying long time and intensive sonication for dispersing them, the resulted suspension is not stable and MWCNTs immediately precipitate from the solvent. Therefore, this property negatively affects the reproducibility of the modification process. To overcome this problem, in most works, before suspension preparation step, MWCNTs are functionalized for example with acid treatment, which facilitates their dispersion and stability of the prepared final modifier sample (54-56). Moreover, some polymeric binders such as chitosan (50), nafion (57), poly (vinyl alcohol) (58), polyaniline (41) and electropolymerized polypyrrole (59) have been employed to facilitate the dispersion of the carbon-based materials and to improve the final stability of the modifier thin film. In some cases, modifier is added to the bulk material of the electrode. This way is mostly used for preparation of the carbon paste modified electrodes (60-65). Moreover, covalent bonding with the help of linking agents, chemisorption via self-assembled monolayers, or electrochemically attachment of modifier to the electrode surface are other ways employed for electrode surface modification. Although, graphene oxide (GO) suspension is easily prepared and stays stable for long time, the reduced graphene oxide (RGO) is hardly dispersed and the obtained suspension is not stable. Therefore, modification of the electrode surface with RGO employing chemically synthesized RGO is time consuming and undesirable. However, GO can be electrochemically reduced and attached onto the electrode surface. In addition, composite of various compounds with carbon nanostructures were used for electrode modification, which combined the synergistic properties of different materials in a simple and versatile way while facilitated the control of preparation conditions (60, 62-70).

**Scheme 1:**
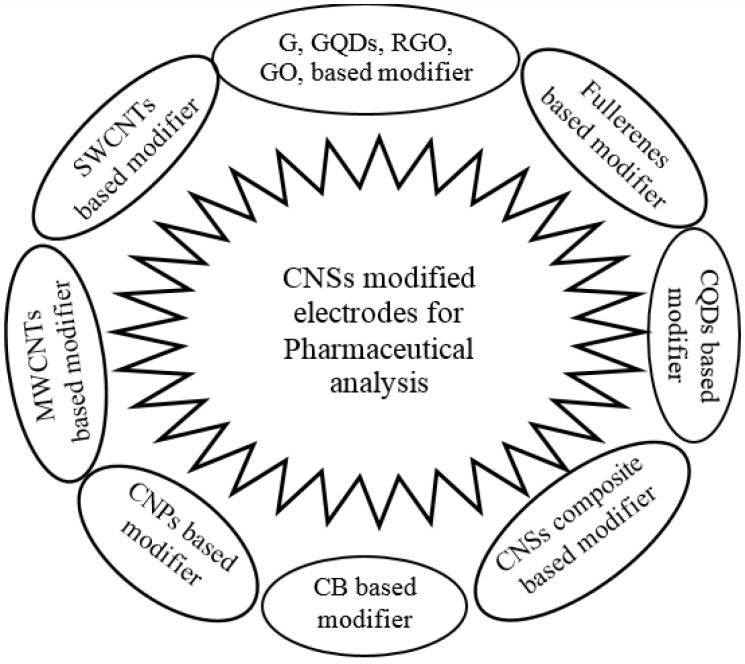
Various carbon nanostructured based modified electrodes (CNSs: Carbon nanostructures; SWCNT: Single walled carbon nanotubes; G: Graphene; GQDs: Graphene quantum dots; GO: Graphene oxide; RGO: Reduced graphene oxide; CQDs: Carbon quantum dots; CB: Carbon black; CNPs: Carbon nanoparticles; MWCNTS: Multi walled carbon nanotubes)

Various types of carbon based nanostructures were employed in electrode modification such as single and multi-walled carbon nanotubes (71-79), graphene oxide and reduced graphene oxide (80-84), graphene (85-87), fullerenes (88), carbon nanoparticles (89-98), carbon black (99-102), and carbon quantum dots (Scheme 1) (103-107). Most of these modifiers improve effective surface area while facilitate the electron transfer between analytes and electrode surface in the electrode-solution interface. In addition, they more effectively adsorbed the analytes and enhanced the electrochemical responses as a result of pre-accumulation on the electrode surface (106-109). Some works dealing with modified electrodes prepared, using carbon nanostructures for pharmaceutical applications aim were listed in Tables 1 to 3.

The combination of CNSs with other nano-structured materials such as metals, metal oxides, and polymers has provided multifunctional hybrid or composites that can significantly improve the sensing properties of the electrochemical sensors. Nano-materials with various type of morphology such as nano-particles, nano-wiers, nano-ribbons, nano-spheres are integrated with CNSs. The prepared heterogeneous nanostructures show outstanding promising potential in sensing applications.

Nanocomposites of Au nanoparticle coated β-cyclodextrin functionalized RGO were synthesized by Pham *et al.* (110). They modified the GCE surface by a thin film of synthesized nano composite and employed it for ciprofloxacin determination. They successfully achieved the detection limit of 2.7 nM by employing differential pulse voltammetry. Recently, He *et al.* constructed an electrochemical sensor based on Cu_2_O-reduced graphene oxide nano-composite (Cu_2_O-RGO) employed for dopamine (DA) analysis (111). A wide range of 0.01 to 80 µM with low detection limit of 6.0 nM was resulted for DA determination. Also, about 204 and 144 mV of peak to peak separation were observed between oxidation peaks of ascorbic acid (AA)-DA and DA-uric acid (UA), respectively. Therefore, analysis of DA can be performed without interference of AA or UA. In another attempt, Nafion/carboxylated-MWCNTs nanocomposite was used for modification of the GCE surface (112). This sensor was applied for timolol maleate (TM) determination. Linear range of 1.0 nM to 20 µM coupled with 0.7 nM detection limit was resulted in analysis evaluation. High selectivity of the prepared sensor toward TM provided its accurate determination in eye drop, urine and water samples without interference of other organic or inorganic compounds. Sharma *et al.* reviewed the surface modified electrodes employed for serotonin detection (113). They reported that various nanocomposite of carbon nanostructures have been used for serotonin sensor construction. For example, a nanocomposite of poly(p-aminobenzene sulfonic acid), (poly-P-ABSA), MWCNTs, and chitosan were prepared and employed for surface modification of the GCE. Here, the electrostatic interactions between negatively charged poly-P-ABSA and positively charged chitosan and special structure of CNTs formed a conductive and stable thin film on the GCE surface. They attributed high conductivity, electrocatalytic activity and adsorption ability properties for carbon nanostructured based composites that effectively improve the sensing ability. The electrochemical behavior of isoxsuprine (ISOX) was evaluated using a GCE modified with nanocomposite of MWCNTs decorated with Ag nanoparticles (114). Two oxidation peaks were observed for ISOX revealing two steps irreversible oxidation. Adsorption of ISOX molecules on the modifier film improved the sensitivity of the electrochemical analysis. High repeatability and reproducibility in response to ISOX beside resulted low detection limit of 12.0 nM revealed applicability of the introduced procedure for ISOX analysis. A flexible screen-printed modified electrode based on electrochemically RGO-CB nano-composite has been employed for detection of dopamine, epinephrine, and paracetamol (115). The hydrophilic surface having high amount of defect sites enhanced the accumulation of target analytes onto the electrode surface, which sensibly improved the current response. This sensor was able to do simultaneous electrocatalytic analysis of three mentioned analytes with very low detection limit. These results confirm the outstanding electrochemical properties of modified electrode based on composite of CNSs.

**Table 1:** Comparison of figures of merit of MWCNTs modified electrodes used in pharmaceutical analysis

**Modified electrode**	**Analyte**	**Method**	**LR** a **(μM)**	**DL** b **(nM)**	**Reference**
P-Dopa/ MWCNTs-COOH/GCE	Metronidazole	DPV	5-5000	250	
MWCNT/PMB/AuNP/GCE	Nevirapine	DPASV^e^	0.1-50	53	
MWCNTs/GO/pyrogallol/GCE	Omeprazole	DPV	0.0002-100	0.01	
Functionalized MWCNTs/GCE	MebeverineHydrochloride	SWAdASV^f^	0.0005-0.035	0.13	
g1-M-3-OITFB/ZnO/CNTs/CPE	Raloxifene	DPV	0.08-400	40	
Poly(o-anisidine)/CNTs/GCE	Mebendazole	DPV	1-35	400	
3DG-CNTN/GCE	Methotrexate	DPV	0.7-199	70	

a Linear range.

b Detection limit.

c Polydopamine/carboxylic multi-walled carbon nanotubes.

d MWCNs/poly(methylene blue)/gold nanoparticle.

e Differential pulsed adsorptive striping voltammetry.

f Square wave adsorptive anodic striping voltammetry.

g 1-methyl-3-octylimidazolium tetrafluoroborate/ZnO/CNTs nanocomposite/carbon paste electrode.

h 3D graphene–carbon nanotube network.

**Table 2:** Comparison of figures of merit of GO, RGO and Gr modified electrodes used in pharmaceutical analysis

**Modified electrode**	**Analyte**	**Method**	**LR** a **(μM)**	**DL** b **(nM)**	**Reference**
GNS-CNTs/MoS2/GCE^c^	Dopamine	DPV	0.1-100	50	
PP3CA/ERGO/GCE^d^	Breast Cancer (BRCA)	DPV	10 fM–0.1	3 fM	
AuNP-rGO-CS/GCE^e^	Methylparaben	SWV	0.03-1.3	13.77	
AuNP-rGO/GCE	Hesperidin	Amperometry	0.05-8	8.2	
Graphen/Carbon paste electrode	Promazine	SWV	0.1-8	8	
Gr nanoplatelets-carbon nanofibers/GCE	Nepafenac	AdSSWV	0.25-15	63	
Graphene/TiO2/V2O5/CPE	Chlorpromazine	DPV	0.033-85	5.3	

a Linear range.

b Detection limit.

c Graphene nanosheets and multiwalled carbon nanotubes.

d Poly pyrrole-3-carboxylic acid/electrochemically reduced GO/GCE.

e Au NPs/RGO/Chitosan/GCE.

f Adsorptive stripping square-wave voltammetry.

**Table 3:** Comparison of figures of merit of CNPs, carbon black and CQDs modified electrodes used in pharmaceutical analysi

**Modified electrode**	**Analyte**	**Method**	**LR** ^a^ **(μM)**	**DL** ^b^ ** (nM)**	**Reference**
Activated CNPs/CPE	Naproxen	DPV	0.1-120	23.4	
CNPs/SDS/CPE^c^	Dopamine	DPV	0.1-100	120	
CNP/GCE	Acetaminophen	DPV	0.1-100	50	
	Tramadole		10-1000	500	
CNP/GCE	Ractopamine	DPV	2-30	0.2	
Nanocellulose/CNP/GCE	Metoclopramide	LSV	0.06-2	6	
Carbon nanofiber/CNP/GCE	Folic acid	DPV	0.1-10	-	
Melamine/CNPs/GCE	Raloxifene	DPV	0.4-2	10	
TiO_2_/Nafion/CNP/GCE	Dobutamine	ASDPV	0.006-1	2	
CB/Ag/PEDOT,PSS/GCE	Paracetamol	SWV	0.62-7.1	12	
	Levofloxacin		0.67-12	14	
CB/GCE	Estradiol	Ampreometry	0.15-3.5	92	
CB/GCE	L-cysteine	Chronoamperometry	50-700	45.87	
CB/GCE	Ethinyl Estradiol	DPV	0.25-3	0.13	
CQD/GCE	Dopamine	LSV^e^	0.19-11.81	2.7	
PBG/CQDs/GCE^f^	Guanine	DPV	0.5-142	16	
	Adenine		0.3-130	26	
β-CD/CQD/GCE^g^	Dopamine	DPV	4-220	14	
	Tryptophan		5-270	16	

a Linear range.

b Detection limit.

c Carbon NPs/Sodium dodecyl sulfate/Carbon paste electrode.

d Carbon black/Ag NPs/PEDOT:PSS/GCE.

e Linear sweep voltammetry.

f Poly(bromocresol green)/CQD/GCE.

g Poly(β-cyclodextrin)/CQD/GCE.

**Table 4:** Comparison of figures of merit of modified electrodes used for dopamine analysis

**Modified electrode**	**Method**	**LR** a **(μM)**	**DL** b **(nM)**	**Reference**
GNS-CNTs/MoS2/GCE	DPV	0.1-100	50	
Chitosan-Graphene	DPV	1-24	1000	
Graphene	DPV	4-100	2064	
MWCNT/GONR^d^	AmperometrY	0.15–12.15	80	
GQD/GSPE^e^	DPV	0.1-1000	50	
Eox-SWCNT/PET film electrode^f^	DPV	1.5-30	510	
CB-ERGO/SPCE^g^	SWV	4.9-19	410	

a Linear range.

b Detection limit.

c Graphene nanosheets-MWCNTs/molybdenum sulfide. flowers/GCE.

d Graphene oxide nanoribbons.

e Graphene quantum dots graphite screen-printed electrode.

f Electrochemically oxidized single-walled CNT/poly(ethylene terephthalate) electrode.

g carbon black- electrochemically reduced graphene oxide composite modified screen printed carbon electrodes.

For analysis of some certain drugs and pharmaceutical compounds the modified electrodes based on various types of carbon nano-structures were developed. For example, MWCNTs and SWCNTs, CNPs, GO, RGO and also carbon black or carbon quantum dots were employed for electrode modification, used in Dopamine (DA) analysis (80, 115 and 116-120), Table 4. Notably, due to high surface area and good conductivity of the mentioned carbon nanostructures, the modified electrode only based on one type of carbon nanostructure provided almost same sensitivity and linear range of determination or limit of detection for DA. However, as mentioned in Table 4, employing hybrid or composite materials of carbon nanostructures effectively improved the linear range and lowered the detection limit of DA analysis.

*Advantages and limitations in clinical complex samples bioanalysis*

Even though, carbon nanostructures based modifiers significantly have improved the analysis sensitivity (28, 47 and 77), as demonstrated, they can respond to the wide range of compounds. Therefore, in most cases they are unable to discriminate between responses of compounds with similar electro-active functional groups in their structures, especially in case of biological fluids which have very complex nature, due to including a wide range of organic and inorganic compounds. For example, as reported, carbon based modified electrodes can effectively improve the response of dopamine, epinephrine, norepinephrine, and other similar compounds. Notably, as the main functional group that undergoes redox reaction in their compounds is quinone - hydroquinone part, discrimination between their responses is not easy and employing carbon nanostructure as electrode modifier alone cannot provide sensible selectivity between them. Therefore, their accurate simultaneous quantification in a mixture solution such as human blood or urine sample is not possible. As reported, addition of other materials such as metal (22, 84), metal oxides (75, 87), organometallic nano-materials (109, 121), and polymeric compounds (71, 73, 76, 104 and 105) and so on to the modification procedure beside the carbon nanostructures can overcome this problem.

## Conclusion

Electrochemical methods employing carbon-based nanostructures can be efficiently employed for pharmaceutical and clinical analysis. However, to improve the selectivity and to access to more sensitivity, it is recommended to apply mixture, hybrid, or composite of these nano-materials with other organic or inorganic compounds. Therefore, it is possible to benefit synergetic or enhancement effect of them together. So, we can access to selective determination of active ingredients of drugs in both bulk pharmaceutical formulation or after administration in biological fluid of the patients’ bodies.
